# Maintaining Transparency of a Heated MEMS Membrane for Enabling Long-Term Optical Measurements on Soot-Containing Exhaust Gas

**DOI:** 10.3390/s20010003

**Published:** 2019-12-18

**Authors:** Luke M. Middelburg, Mohammadamir Ghaderi, David Bilby, Jaco H. Visser, Guo Qi Zhang, Per Lundgren, Peter Enoksson, Reinoud F. Wolffenbuttel

**Affiliations:** 1 Department of Microelectronics, Faculty of EEMCS, Delft University of Technology, Mekelweg 4, 2628CD Delft, The Netherlands; g.q.zhang@tudelft.nl; 2 Chalmers University of Technology, Department of Microtechnology and Nanoscience, EMSL, Kemivågen 9, 412 58 Gothenburg, Sweden; mohammadamir.ghaderi@chalmers.se (M.G.); per.lundgren@chalmers.se (P.L.); peter.enoksson@chalmers.se (P.E.); R.F.Wolfenbuttel-1@tudelft.nl (R.F.W.); 3 Research and Advanced Engineering, Ford Motor Company, Dearborn, MI 48121, USA; dbilby@ford.com (D.B.); jvisser@ford.com (J.H.V.)

**Keywords:** optical automotive instrumentation, optical MEMS, heated silicon carbide window, suspended membranes, on-board diagnostics, surface regeneration from soot deposits, thermophoretic repulsion of soot

## Abstract

Ensuring optical transparency over a wide spectral range of a window with a view into the tailpipe of the combustion engine, while it is exposed to the harsh environment of soot-containing exhaust gas, is an essential pre-requisite for introducing optical techniques for long-term monitoring of automotive emissions. Therefore, a regenerable window composed of an optically transparent polysilicon-carbide membrane with a diameter ranging from 100 µm up to 2000 µm has been fabricated in microelectromechanical systems (MEMS) technology. In the first operating mode, window transparency is periodically restored by pulsed heating of the membrane using an integrated resistor for heating to temperatures that result in oxidation of deposited soot (600–700 °C). In the second mode, the membrane is kept transparent by repelling soot particles using thermophoresis. The same integrated resistor is used to yield a temperature gradient by continuous moderate-temperature heating. Realized devices have been subjected to laboratory soot exposure experiments. Membrane temperatures exceeding 500 °C have been achieved without damage to the membrane. Moreover, heating of membranes to ΔT = 40 °C above gas temperature provides sufficient thermophoretic repulsion to prevent particle deposition and maintain transparency at high soot exposure, while non-heated identical membranes on the same die and at the same exposure are heavily contaminated.

## 1. Introduction

Full characterization of exhaust gas is essential for meeting increasingly strict automotive emission requirements. State-of-the-art exhaust gas sensing is based on devices in direct contact with the gas flow, such as electrochemical sensors for the measurement of gas composition [1] and electrical conductance (conductometric) sensors for particulate matter (PM) [2]. The next generation of sensors for testing compliance with emission requirements should be suitable for the measurement of soot particle number concentration (PN) in relation to their size distribution, which is difficult to achieve when using state-of-the-art conductometric soot sensors [3]. Optical techniques provide a more robust approach in the harsh environment of the soot-containing gas in the tailpipe of the combustion engine and would enable the non-contact collection of more information as compared to currently used techniques, provided that optical access is maintained. Deposition of soot on the window that provides a view into the tailpipe needs to be prevented. The reduced light transmission through the window over time, due to the build-up of such light-absorbing layers of contaminants in its path of propagation, has hindered the implementation of otherwise potentially promising optical approaches for measuring exhaust gas properties [4]. Gas properties of interest, such as composition, can be analyzed using absorption spectroscopy and soot particle size distribution using angular scattering spectroscopy. Results of short-term exhaust gas composition measurement based on absorption spectroscopy have been reported using glass-fiber probes for passing light into the exhaust system [5,6,7,8]. An optical window is also a functional part of a probe for Laser-Induced Incandescence (LII) studies on soot particles [9]. However, the duration of reported tests on actual exhaust gas does not exceed 30 min [10]. Therefore, approaches for restoring or maintaining window transparency are an essential prerequisite for the successful introduction of optical techniques for long-term, in-situ monitoring of exhaust gas emissions, as will be required by regulations.

The wall of the exhaust pipe is the colder part during normal operation of the combustion engine. Combustion results in hot exhaust gas and, consequently, in temperature gradients in the exhaust pipe, with a descending maximum temperature at the center of any cross-sectional area along the axial direction and a minimum at the wall. The radial temperature gradient causes the thermophoretic force that pulls the soot particles in the gas flow towards the colder inner wall surface. Thermophoresis (or thermo-migration or thermo-diffusion) can be explained by referring to the temperature dependence of the Brownian motion of gas molecules, which increases with temperature. In a highly simplified interpretation, gas molecules in between the particle and the center of the flow channel vibrate more vigorously as compared to those in between the particle and the colder wall, thus yielding a net force pushing the particle towards the wall. A more fundamental analysis of this phenomenon can be found in the literature, for instance by Bakanov [11].

Thermophoresis is actually part of the operating mechanism of the conductometric PM sensor. A direct current (DC) bias voltage of about 45 V is applied across two interdigitated electrodes (IDEs). Soot particles in the exhaust gas flowing along the sensor surface are pulled towards the sensor surface, because of the combined effect of the thermophoretic pulling force (due to the colder wall surface on which the sensor is mounted) [12] and the electrophoretic force (due to the electrostatic field) acting on charged particles [13]. As a result, soot particles are deposited and form dendrites (connected strings of particles) on the sensor surface, eventually bridging the two IDEs. Since carbon is electrically conductive, such a dendrite forms an electrically conductive path. The resulting decrease in resistance is used as a measure of PM [2].

In this work, a window composed of an optically transparent intrinsic poly-SiC membrane with a diameter ranging from 100 µm up to 2000 µm has been fabricated in Si-based microelectromechanical systems (MEMS) technology. N-doped poly-SiC heaters are fabricated on top of the membrane and two modes of operation are investigated for maintaining transparency. In the first mode, window transparency is periodically restored by pulsed heating of the membrane using the integrated resistor for heating to temperatures that result in oxidation (burning) of deposited soot (600–700 °C). In the second mode, the membrane is kept transparent by continuous heating using the same integrated resistor to result in a temperature increase, ΔT, of typically 40 to 100 °C relative to the temperature of the surrounding gas it is exposed to (typically at 400 °C). The resulting thermophoretic force repels airborne soot particles in close proximity of the surface and prevents deposits on the membrane. Combined operation is envisaged, using mode 2 continuously for minimizing deposition and mode 1 at regular intervals for the removal of any remaining deposits. A typical system includes several windows mounted on the wall of the exhaust pipe, with some providing access of a light beam that is intended for interacting with the gas, and others for ensuring transmission of the resulting information-carrying modulated light to a detector.

Integrated resistors are used in the conductometric PM sensor for regularly heating of the sensor up to a temperature of about 600 °C for periodically oxidizing (burning) the soot, which is referred to as ‘regeneration of the surface’ [12]. This known mechanism for surface regeneration can in principle be directly implemented for periodically restoring window transparency. However, care should be taken with any remaining thin films of, for instance, oil. In the case of light with a wavelength component λ, a residue with an (optical) thickness in the range λ/10–λ/2 may cause interference that significantly affects the optical spectral transmission, while such a film may have no noticeable effect on proper operation of the conductive PM sensor. Therefore, the presence and effect of such films need to be investigated for an assessment of the effectiveness of mode 1 operation for optical regeneration of the membrane surface.

Thermophoresis has been explored in conductometric PM sensors as a means of sensitivity control. A reduced sensitivity with increased temperature was confirmed [12]. Thermophoresis can also be exploited for keeping a selected surface area clean of deposits by intentionally locally heating of the sensor surface up to temperatures slightly above the exhaust gas temperature for repelling airborne particles. The results of the utilization of thermophoresis for maintaining a transparent window is presented (referred to here as mode 2 operation).

Heating of membrane structures fabricated in silicon MEMS technology has been extensively reported in the literature and usually involves a material combination of (poly)silicon, silicon-oxide and silicon-nitride with integrated resistive heaters. Applications are in devices that require only a few degrees of temperature above ambient temperature, such as thermal flow sensors [14], while heating is used in thin-film SnO_2_-based gas sensors for setting the surface temperature for maximum sensitivity [15]. Hot-wire blackbody emitters have been fabricated for use in infrared (IR) optical applications [16]. Usually, the heated devices are fabricated on a membrane or ceramic substrate to limit heat loss due to thermal conduction to ambient and thus ensure power-efficient operation.

The application of a regenerable optical window presented here imposes specific requirements. Firstly, the exhaust environment is generally considered a harsh environment, both from the thermal and the chemical perspective. Heating to membrane temperatures of 700 °C is required for restoring window transparency by oxidization of deposited soot. Secondly, the membrane material should preferably be transparent over the spectrum between the near-ultraviolet (UV) (220 nm) and mid-IR (5 µm). A conventional membrane material, such as SiO_2_, does satisfy this requirement, while other materials, such as Si_3_N_4_, are less suitable. Poly-silicon (poly-Si) can only be considered a transparent material in the IR (and highly absorbing in the visible spectral range). The electrically conductive layers used for heater fabrication should, preferably, also be transparent.

Silicon-carbide (SiC) is a highly suitable building material for both the transparent membrane and the heater structure. SiC has numerous advantages over mainstream silicon technology. Highly relevant to the application of regenerable optical window considered here are its high chemical inertness, optical properties and high melting temperature, but its high thermal conductivity, high critical E-field, high Young’s modulus, high radiation hardness and high acoustic velocity are significant advantages in other applications. Moreover, owing to its large band-gap, micro-electronic components can be operated at elevated temperatures, even beyond 500 °C [17,18]. SiC-based technology is already considered at a mature stage for the fabrication of power electronics, but developments in the field of harsh environment MEMS sensor development are ongoing [19]. Indeed, SiC has already been considered in the literature for the fabrication of hot-wire emitters operating at high temperatures [20].

Among the different forms and polytypes available, a common candidate in MEMS device processing is 3C-SiC in polycrystalline form. The advantages of this material are that it can be conformally deposited in a low-pressure chemical vapour deposition (LPCVD) furnace, while in situ doping is applied to control the doping level and residual stress level of the resulting poly-crystalline layer. The potential of the integration of the poly-SiC material deposition with silicon substrates, along with the silicon technology portfolio, is exploited in this work. Details on the deposition and characterization of the layers as used in this work are reported in the literature [21].

Intrinsic SiC can be considered a low-loss optical material in the spectral range between 400 nm and 2 µm. However, depending on doping concentration, doped SiC can be used as an optical material of reasonable performance in the spectral range between 400 nm and 800 nm. This restriction, as compared to the desirable UV-to-IR, limits the application to the visible. The index of refraction, *n* and extinction coefficient, *k*, of SiC and wavelength dependencies thereof depend on the specifics of the deposition.

Design considerations are presented in the next section, followed by fabrication. Results of experimental validation are shown in Section 3. Finally, conclusions and directions for further research are given.

## 2. Materials and Methods

### 2.1. Design of the SiC Windows

**Figure 1:**
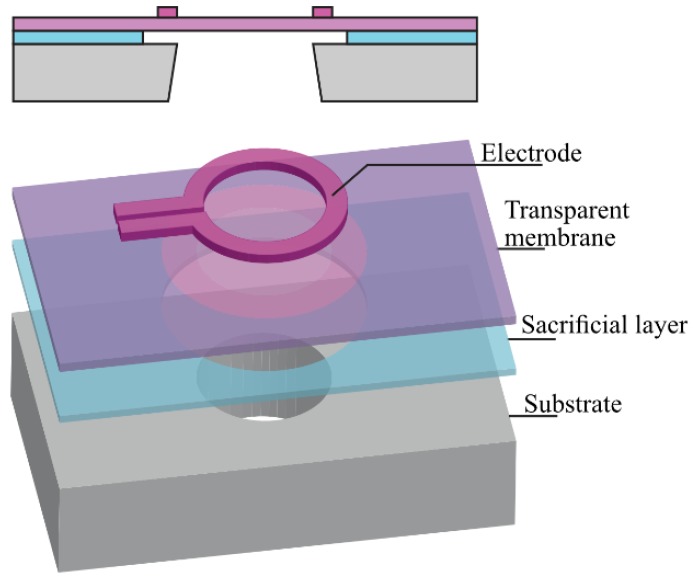
Schematic view of the basic structure of the transparent membrane and the heater. Note that the metallization is not included in this view.

The conceptual structure of the optical window with the components required for restoring or maintaining transparency by (a) oxidation of deposits and (b) generating thermophoretic forces is shown schematically in Figure 1. The membrane layer and the ring-shaped heater can be clearly distinguished.

The structure is basically composed of a suspended optically transparent membrane. An electrically conductive layer on top is patterned into a resistor and can be used for heating of the membrane. The heater can be used for both for the surface regeneration in mode 1 and the thermophoretic repulsion in mode 2. The heater should, preferably, be optically transparent for effective use of the entire membrane area as a window. In case of significant differences in optical transmission between the membrane- and the heater material, or differences in their wavelength dependencies over the spectral range considered, a design that blocks the light at the position of the latter is to be used to avoid a wavelength-dependent average transmission throughout the window area. The reduced area that is available as window due to opaque electrodes and heater is sometimes specified in optics in terms of the ‘fill factor’ (i.e., the fraction of the area that can actually be used). Bulk micromachining of the silicon substrate in a deep reactive ion-etching (DRIE) process is applied for fabrication of the dielectric membrane that is to function as the optical window.

The dimensions of the membrane result as a compromise between the minimum aperture required for passing a practical light beam, and the maximum diameter that can be sustained when considering residual stress in the layers that comprise the membrane and its thickness. The absolute value of the tensile stress increases with increasing dopant concentration in the poly-SiC layers considered. A small to moderate tensile stress is desired in order to achieve a taut (i.e., flat, non-buckled) membrane. The SiO_2_ layer functions as the landing layer for the through-wafer etch, and its thickness was chosen to be 2 µm. In order to yield a membrane with minimal optical extinction, its thickness should be kept to a minimum. On the other hand, a larger thickness results in a higher mechanical strength, which is required considering the application in an automotive exhaust environment. The membrane layer thickness was designed to be 500 nm. The diameter of the windows ranges between 50 µm and 2000 µm.

A uniform temperature profile is desirable for optimal operation of the transparent window, with minimum membrane area occupied by the heaters. Moreover, power efficiency should be considered. Limiting heat diffusion from the membrane to the surrounding rim implies that the outer part of the heater should be at a certain distance from the rim, which makes a compromise between a uniform temperature profile and low power dissipation inevitable. Three-dimensional finite element analysis (FEA) using measured material properties and considering realistic flow conditions was applied to analyze the geometry of the heater and membrane. The layout design was optimized for uniform temperature at a given power dissipation.

**Figure 2:**
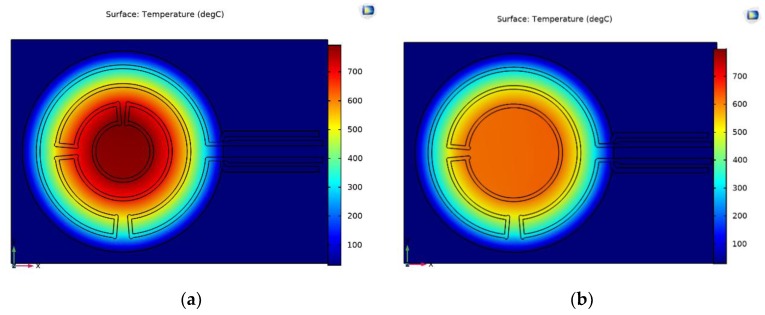

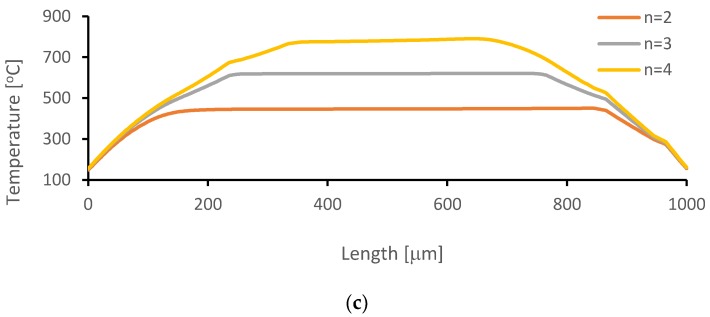
(**a**) Lateral temperature distribution on a MEMS window for 4 heater loops and (**b**) 3 loops at 3 W; (**c**) Temperature profile along the diameter of the membrane at 3 W injected power for a different number of heater loops.

LPCVD is used for deposition of an intrinsic SiC film and a highly doped SiC film. The sheet resistance of the deposited films was measured using the four-point Kelvin measurement. Based on the high difference between the resistivities of the intrinsic SiC (about 440 Ω·cm) and the highly doped SiC layer (ρ_HIGH_ = 3.36 × 10^−3^ Ω·cm) it can be calculated that the current densities in these layers differ by three orders of magnitude. Based on the large separation of the electrical bulk resistivity between the intrinsic and the highly doped version of the poly-SiC layer, it was decided that a passivation layer in between the heater and membrane layers is not necessary, thus favoring fabrication convenience. These values were included in the finite element model (FEM) for analyzing the different heater designs at realistic conditions and the results are shown in Figure 2. The heat loss due to conduction in the membrane and the gas and convection through the fast-flowing gas above the membrane were considered, while the radiation loss was estimated to be much smaller than the conductive loss and was ignored. Moreover, the temperature coefficient of resistivity (TCR) of the undoped SiC is not considered in these simulations. These assumptions limit the validity of the simulations to the lower operating temperature range. The membrane diameter was kept constant at 1000 µm and the temperature profile of a heater with a different number of turns is shown. The width of the wires was 20 µm.

At 3 W constant injected power, the structure with more heater loops generates a higher peak temperature at its center. Most implementations are based on four-loop heater designs, for enabling a peak temperature in excess of 700 °C for more than 40% of the membrane area in case of a diameter exceeding 1000 µm.

**Figure 3:**
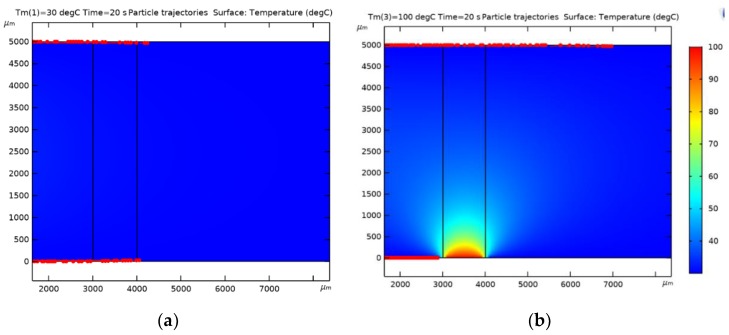
Simulations results on soot deposition distribution with a heater geometry on top of a window membrane of 1000 µm diameter at a position between 3000 μm and 4000 μm at membrane temperatures of 30 °C (**a**) and 100 °C (**b**).

The propagation of soot and its build-up over time when placed in the flow channel is another important aspect of the membrane design, which can be modelled for the different operational conditions. A 5 mm diameter flow channel was used in the simulations, while the gas and the wall of the flow channel are at 50 and 30 °C, respectively. At the given dimensions and flow, laminar flow can be assumed, which limits the sensitivity of the results to variations in the actual diameter of the flow channel. The presence of the heated window results in a temperature distribution along the channel, which is used for calculating the temperature gradient induced thermophoretic forces that are acting on particles. The soot particles are carried in the laminar flow with a velocity that varies from close to zero in the boundary layer nearby the walls of the channel to its maximum value at the center. In the simulations, deposition is assumed to take place when the velocity of the particle in the boundary layer of the flow becomes zero due to the thermophoretic dragging force. Interactions between particles are not considered in these simulations. Figure 3 shows the temperature profile and the resulting build-up of soot along the channel, as modelled in a 2D cross-section, with soot particles entering from the left and the membrane area position between 3000 µm and 4000 µm at the horizontal axis.

Figure 3a shows a uniform deposition of soot particles on the two opposite walls of the flow channel. The simulations do not consider any local turbulence in the flow that replenishes particles in the boundary layer of flow by mixing from the center, which results as the apparent depletion of soot particles in the flow after 4000 µm length. As shown in Figure 3b, the thermal gradient caused by the presence of the heated window prevents particles from deposition on the window. The results of the modelling also reveal that the repelling thermophoretic force acting on approaching particles not only repels particles from the membrane area, but also results in the build-up of deposits before particles reaching the heated window area. The thermophoretic repulsion opposes the motion of particles approaching the membrane along the wall, thus is experienced as an additional dragging force with increased local deposition. Figure 3b also shows that the number of particles deposited on the opposite wall downstream of the membrane is larger compared to the unheated case. This can be explained by the fact that the thermophoretic force exerts an outward force to the particles. Such effect could only be expected in a laminar flow in which turbulent forces that could counteract the thermophoretic force at long distances are not present.

### 2.2. Fabrication

The intrinsic SiC layer is to be used as optical window, while the highly doped layer is patterned to form the heater structure. The poly-SiC is deposited by LPCVD where the precursor gas flows can be tuned. The ratio between the two precursor gasses is denoted by the Gas Flow Ratio (GFR). The GFR essentially determines composition of the SiC layer and its physical properties. Additionally, ammonia can be added during deposition, resulting in doping with nitrogen (n-type). For the intrinsic (undoped) carbide, the GFR is chosen such that a maximum resistivity is obtained. More details on the deposition of the poly-crystalline SiC layers are reported by Morana et al. [21].

Practical implementation of SiC as a material for a transparent window requires a wavelength-independent index of refraction, *n*, for wideband index matching to air or dielectrics such as SiO_2_ that are often used for surface coating, and low values for the extinction coefficient, *k*, for low-loss optical transmission. In preliminary experiments the optical properties of intrinsic and doped SiC layers are measured using variable-angle spectroscopic ellipsometry (J.A. Woollam M2000) on 1000 nm-thick layers on 500 nm SiO_2_ that is thermally grown on Si. Subsequent modelling in WVASE (J.A. Woollam) is used to derive the optical properties over the entire visible spectral range between 400 nm and 650 nm as index of refraction, *n* = 2.4 ± 0.2 and extinction coefficient, *k* < 0.1.

**Figure 4:**
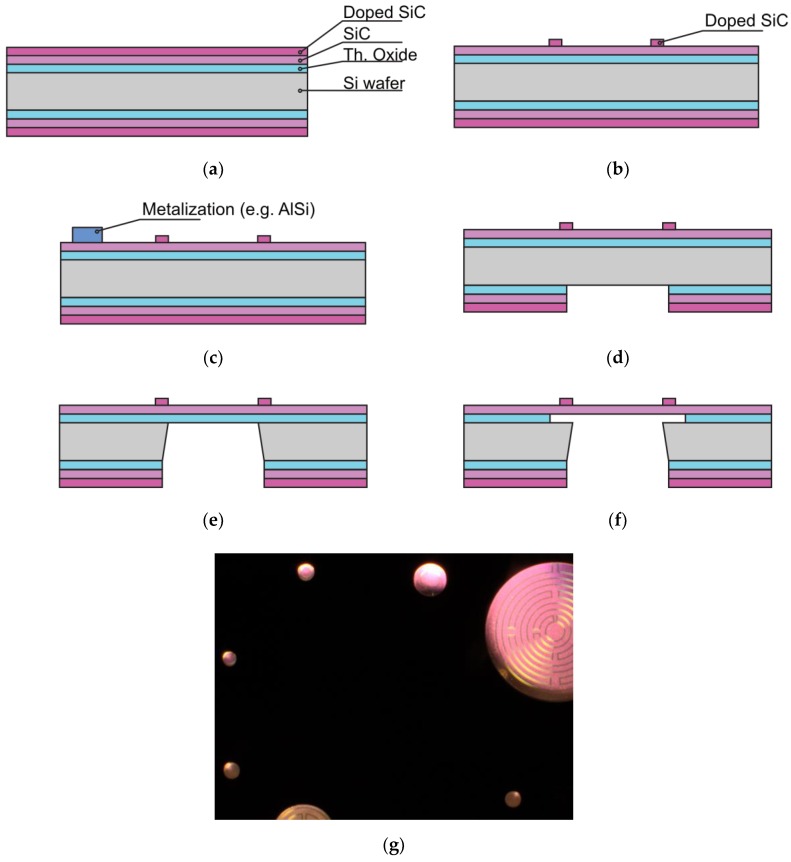
(**a**–**f**) Schematic flow of the fabrication process; (**g**) inspection with an optical microscope at backlight illumination of membranes which are etched-through and opened.

Device fabrication starts with the growth of 2 µm of SiO_2_ by thermal oxidation of a silicon wafer, which is intended as etch-stopping (landing) layer during through-wafer etching and hardmask at the backside (BS) for definition of the DRIE cavity etching. In addition, this layer provides some thermal isolation between the substrate and the subsequently deposited poly-SiC layers. After thermal oxidation, two poly-SiC depositions by LPCVD followed, as shown in Figure 4a, using the already characterized intrinsic and highly doped poly-SiC layer. The residual stress for the intrinsic layer was measured to be 65 MPa and for the highly doped layer 503 MPa. Such moderate tensile stress levels ensure flat membranes without buckling.

The next step is the coating of the frontside (FS) of the wafer with 4.0 μm AZ3027 photoresist (PR) and applying lithography. A timed etch was used for the definition of the heater structure out of the doped poly-SiC layer, as shown in Figure 4b. A DRIE etcher (SPTS Rapier i2L) in inductively coupled plasma (ICP) mode was used. The etch recipe is based on an SF_6_/O_2_ mixture at 0 °C to enhance selectivity between the PR and poly-SiC.

After the heater structures were formed, standard wafer cleaning was performed followed by metallization. For five wafers 500 nm AlSi (1%) was deposited and on another set of five wafers 500 nm pure titanium (Ti) was deposited. Both depositions were carried out at 350 °C to yield the best metallization quality and low contact resistance. Lithography was performed and the patterned AlSi (1%) and Ti were etched using a plasma etcher (Trikon Omega) at 25 °C, after which the wafers were stripped cleaned reaching the stage as shown schematically in Figure 4c.

The hardmask on the BS, which is composed of the thermal oxide layer and the two poly-SiC layers, was opened in phases. First backside lithography is performed and the poly-SiC was etched with an Omega ICP etcher. The thermal oxide was subsequently opened using wet-chemical etching with 1:7 buffered hydrofluoric (BHF) etch solution (Figure 4d). The frontside of the wafer was coated with photoresist for protection of the metallization. Etching through the wafers was done with an SPTS Rapier DRIE etcher (Figure 4e). Because both poly-SiC and thermal oxide are transparent in the visible range, visual inspection using an optical microscope with backlight illumination can be used to see which of the membranes are open, see also Figure 4g.

**Figure 5:**
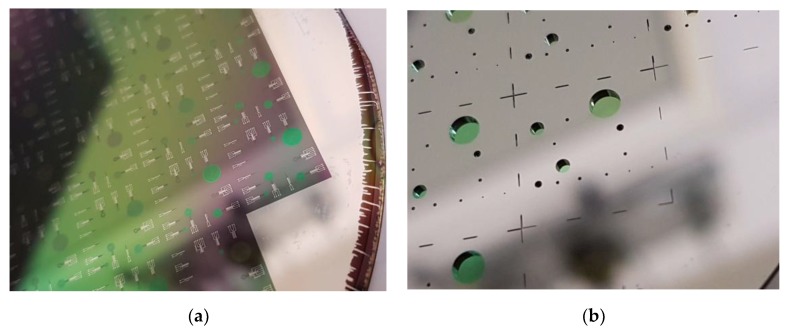
Membranes with integrated heaters and Al interconnect after release in: (**a**) frontside view and (**b**) backside view.

Due to the stress mismatch between the 500 nm poly-SiC membrane (moderately tensile) and the 2 μm thick silicon oxide landing layer (compressive), the membranes are buckled. Therefore, it is important to remove the thermal oxide used as landing layer as soon as possible after through-wafer etching. For the wafers with AlSi (1%) interconnect (IC), the oxide etching was achieved in vapor HF. Four cycles of 300 s each were used to give a successful result as shown in Figure 4f. Figure 5a,b shows respectively the frontside and the backside of the devices after the silicon oxide has been removed. The wafers with aluminum IC were spray-coated after this release step to enable the removal of the dicing foil from the thin membranes in an acetone rinse during final dicing and packaging.

**Figure 6:**
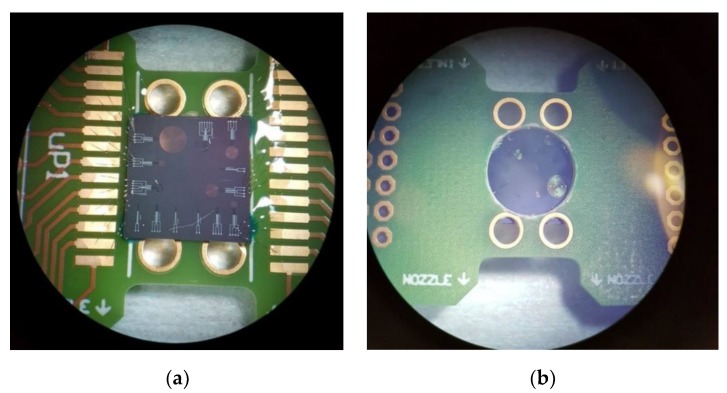
Dies bonded on a test printed circuit board (PCB): (**a**) frontside view and (**b**) backside view.

For the wafers with Ti interconnects, wet-chemical etching with 1:7 BHF was used to remove the thermal oxide landing layer, after the FS was protected again by a spray-coated PR layer and subsequent baking steps. Figure 6 shows the result after completion of wafer processing.

Wafers are diced into 10 × 10 mm^2^ dies and subsequently bonded on a printed circuit board (PCB) with a 9.5 mm hole by using thermal curable glue. The dies are aligned in such a way that all membranes on the die can be simultaneously illuminated and inspected, as shown in Figure 6. Initial experimentation is limited to exhaust gas at room temperatures, which a PCB can withstand. Wire bonding with aluminium wire was applied for the chips with AlSi (1%) IC, whereas bonding with gold wire was used for the chips with Ti IC. UV curable glue was applied below, on and around the bonding wires for mechanical support during transportation and measurements.

## 3. Results

**Figure 7:**
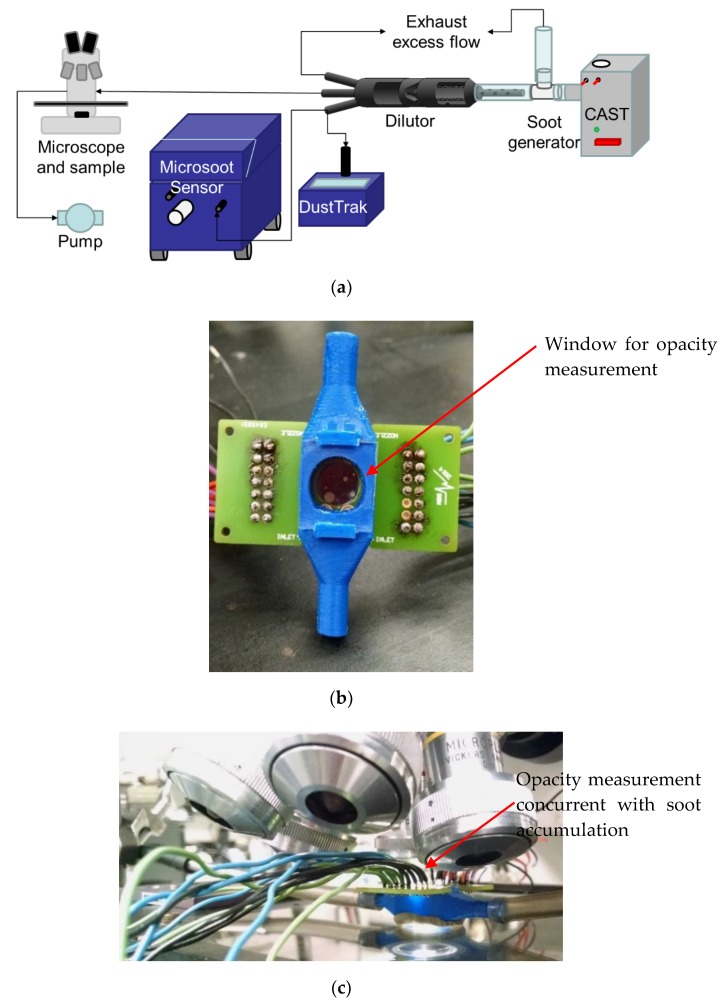
Measurement set-up: (**a**) generating soot for reproducible distribution; (**b**) window in the 3D-printed housing; (**c**) window under inspection during exposure.

Actual validation of the interaction between the fabricated windows with soot-containing gas is carried out by in situ measurements of opacity using an optical microscope and an aerosol conditioning setup (Figure 7a). A Jing MiniCAST 5201c creates a reproducible lognormal soot distribution with a mean geometric diameter near 80 nm by burning propane. A vacuum pump and a mass flow controller are used to pull flow through the 3D printed sample holder housing at a flow rate in the range 100–1000 cm^3^/min. (Figure 7b). The housing constrains the PCB and slide glass to create an optical path for observation of soot deposition through transmission mode optical microscopy. Note that the housing provides a mechanism observing the sample from the normal direction to the surface area, while subject to soot-containing aerosol exposure flowing along an orthogonal path.

Samples were exposed at the backside of the membrane. A Vickers optical microscope fitted with a Dino-Lite eyepiece USB camera was used for periodic imaging with constant exposure levels to enable the observation of soot accumulation on heated and un-heated membranes (Figure 7c). A Keithley 2420 source measurement unit (SMU) was used to drive the heater at constant voltage or constant current and data acquisition is based on Labview. Soot-rich flow was diluted and for verifying the actual soot exposure of our test sample, levels were measured using an AVL microsoot sensor and TSI DustTrak.

The nominal heater resistance at room temperature for the 1000 µm diameter membrane is R_o_ = 36.85 kΩ at 298 K. The heater resistance is correlated with window temperature via oven and infrared camera calibration experiments. The oven calibration used a setup exploiting a Lindberg/Blue M tube TF55035A furnace and an Omega K-type thermocouple, while the DC-impedance was measured simultaneously using a Keithley 2420. The inspection with the infrared camera was done using an FLIR 325sc camera in combination with a 2× zooming lens, while an emissivity coefficient of 0.80 was used. A linear regression of temperature with heater resistance is used to infer the sample temperature in soot deposition experiments where only heater resistance is known. The resulting average TCR (α) over different heater structures equals α = −3289 ± 89 [ppm/°C]. The uncertainty is assumed to be due to non-linearity only.

The objective is to demonstrate restoring of the optical transparence of the membranes by removal of the deposited soot by oxidation at elevated temperatures. It is known from literature that the soot regeneration reaction is an abrupt function of temperature, with the onset to combustion at about 500 °C to full combustion at 700 °C, depending somewhat on operating conditions [22]. The devices were designed for surface regeneration (mode 1) over the entire membrane area. However, the temperature dependence of the free carrier concentration in the intrinsic SiC membrane results in a localized increase of leakage current at the warmer parts of the membrane and, consequently, in a further increase of localized heating. For mode 1 operation, this thermoelectric feedback mechanism results in high temperatures only at localized regions (hot spots) over the window. These hot spots were used here for the surface regeneration experiments. The hot spots result from a higher-than-expected leakage current between the intrinsic SiC layer and the doped SiC at elevated temperatures and this is due to the temperature dependence of the free-carrier concentration in the intrinsic layer. To achieve the uniformly distributed and high-peak temperature profile required for mode 1, dielectric insulation between membrane and heater needs to be included.

**Figure 8:**
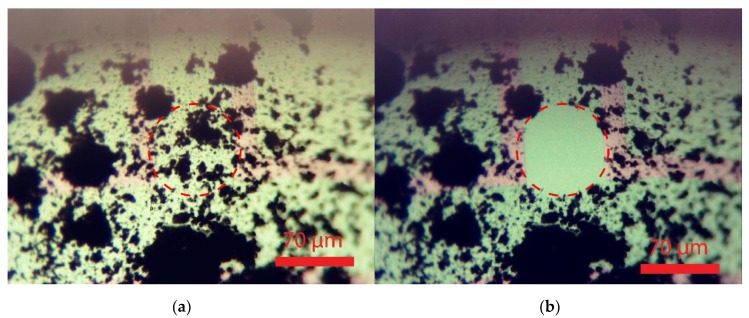
The membrane before (**a**) and after (**b**) the regeneration process, note that the soot is locally removed at the hot spot without permanent deformation of the membrane.

Figure 8a shows a membrane with a considerable soot deposition for testing mode 1. The soot on this membrane is deposited by impaction, rather than by diffusion in the in-line flow soot accumulation setup, by pointing the outlet of the diluted soot rich gas at an angle of approximately 60 degrees to the backside of the membranes. The advantage of impaction is the higher soot deposition rate. The heater current was slowly ramped up until the regeneration process was observed. The result of the regeneration experiment is included in Figure 8b. The regeneration time for reaching the situation shown in Figure 8b from that in Figure 8a is typically 5 min. Power dissipation for a hotspot of about 40 µm is 30 mW, which is equivalent to 6 µW/µm^2^, as compared to 0.95 µW/µm^2^ used in the full-membrane area simulations.

**Figure 9:**
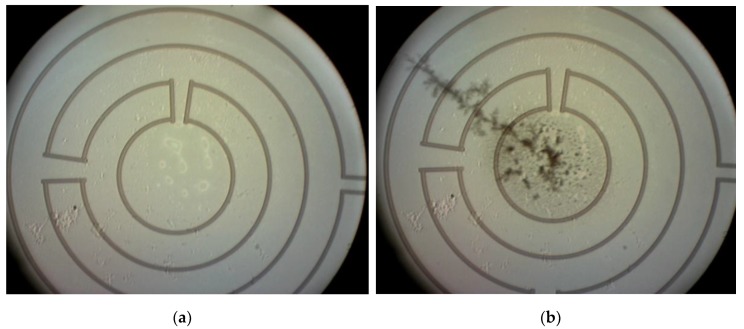
Images of an unheated 1000 µm membrane at: (**a**) *t* = 0 s and (**b**) *t* = 70,700 s.

After successfully restoring local transparency at hot spot locations by soot oxidation, the effect of thermophoretic repulsion as an approach for contamination prevention (mode 2 operation) was investigated. Figure 9a,b shows the images taken from the unheated membrane at *t* = 0 s and *t* = 70,700 s respectively. Aerosol flow was 100 cm^3^/min and soot concentration was between 10–12 mg/m^3^. Soot has been deposited on the backside of the membrane and a large soot dendrite is formed. The soot deposition is by diffusion from a laminar flow, as can be concluded from the soot dendrites in the direction of the flow.

**Figure 10:**
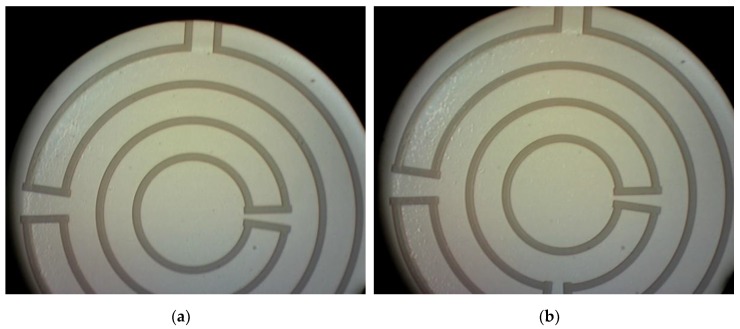
Images of a heated 1000 µm membrane at: (**a**) *t* = 0 s and (**b**) *t* = 70,700 s.

Figure 10a,b shows the images taken from the heated membrane at *t* = 0 s and *t* = 70,700 s respectively. As can be concluded from these images, the heated membrane stays clear from soot deposition in case of a temperature gradient between the membrane and the soot-rich gas of around 35 °C. Although this temperature gradient is small, it is shown to be sufficient for an effective repulsion of soot.

**Figure 11:**
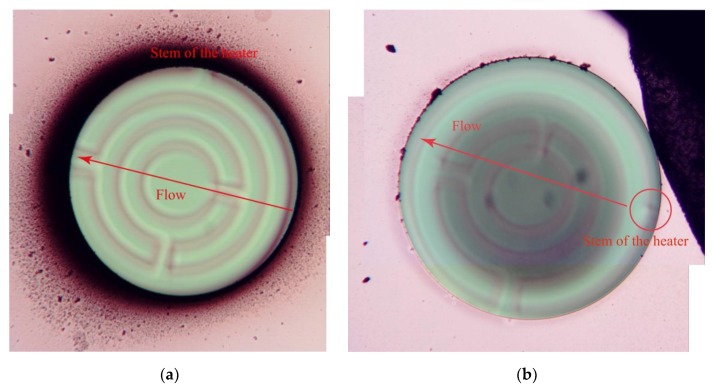
Backside inspection (focal plane on substrate) of two different 1000 µm-diameter windows after the same soot exposure, with the membrane in (**a**) heated and the membrane in (**b**) not heated. While the heated window has remained free of deposition due to thermophoretic repulsion of passing soot particles, significant soot deposition has occurred around the cavity opening. The unheated window has soot deposition on the membrane and a somewhat reduced deposition on the cavity edge as compared to the heated case.

Closer inspection by optical microscopy of the backside of the structure, as shown in Figure 11, reveals a significantly higher amount of soot deposition downstream the soot-containing gas flow and near the heated membrane opening. This effect is most likely to be the result of turbulence at the ridges of the etched gap and is not related to the thermophoretic repulsion. Note that the combined thickness of the wafer and PCB results in a recess of about 2 mm at the membrane position that extends perpendicular to the flow direction, resulting in a turbulent flow close to the window. The reduced local flow velocity at passage of the gap is likely to result in an increased downstream particle deposition.

## 4. Discussion and Conclusions

The MEMS optical membranes in SiC fabricated in this work do provide preliminary validation of their suitability as transparent MEMS platform with a view into the soot-containing exhaust gas. Restoring window transparency by heating at about 600 °C (mode 1 operation) has been essentially demonstrated. Although the mode 1 operation was limited to small hot spots, the results show effective removal of soot from the window, thus restoring its transparency. Moreover, thermophoresis for maintaining a transparent window (mode 2 operation) has been demonstrated for heating to 35 °C above the soot-containing gas temperature. It should be emphasized that these results are preliminary, as the experimentation was carried out using a CAST soot generator for generating a particle flow close to room temperature. High temperature survivability was shown by the regeneration tests, where no permanent material change was observed, nor shifts in the device performance from an electrical or mechanical point of view. The favorable optical properties of SiC have been part of the experimentation, as the imaging of deposits at the backside of the membrane, as presented in Figure 8, Figure 9 and Figure 10, has taken place through the membrane, and thus have been confirmed.

The MEMS heater design was adequate for repeatedly demonstrating the effect of thermophoretic repulsion of soot particles in the exhaust gas. Surface regeneration was demonstrated by oxidation of severe contamination by impacted soot. Regeneration was achieved for a hot spot area of about 1000 µm^2^ in less than 5 min, without any permanent damage or deformation to the membrane. Power dissipation for a hotspot of about 40 µm is 30 mW, which is equivalent to 6 µW/µm^2^, as compared to 0.95 µW/µm^2^ used in the simulations. The difference is mainly due to the small spot diameter and an improved agreement in case of full-membrane heating is expected. Visual inspection showed that transparency was maintained after the regeneration. The occurrence of hotspots during deposition implies that a dielectric isolation layer between the membrane and heater poly-SiC layers is required for a larger and more homogeneously heated membrane.

Future work includes the assessment of the mechanical robustness and chemical inertness of the poly-SiC membranes in an industrial measurement setup and high-temperature packaging of the device. Moreover, more detailed FEA is required to improve the understanding of the soot repulsion and deposition at local turbulence. Also, the effectiveness of thermophoretic repulsion with increased temperature should be validated over a range of temperature differences. Additional mechanisms, such as electrophoresis should be considered. Finally, the optical properties of the membrane, such as the spectral transmission should be measured as a function of membrane temperature.

The 3D printed window package is not suited for high-temperature operation. Consequently, a packaging redesign will be required to allow exposure to actual exhaust gas.

Although the simulation and experimental validation of the thermophoretic effects of the temperature gradient on airborne particles passing by within the short distance of the boundary layer width of the flow are in good agreement, there remain some issues related to the soot deposition outside the heated membrane. These effects are not directly relevant for maintaining a transparent window, but may affect the long-term performance and require further attention.

Effective use of thermophoresis for maintaining transparent windows is ensured only if the window temperature is raised by the intended temperature difference relative to the temperature of the exhaust gas at the position of the membrane. This implies that the local temperature needs to be measured, which can be implemented conveniently by patterning of a temperature-dependent resistor (thermistor) in the same poly-SiC layer used for fabrication of the heater.

Although probably less effective as compared to thermophoresis, electrophoresis may have merit as an auxiliary mechanism in mode 2 operation for providing an improved performance in repelling large and charged particles in particular, when combined with thermophoresis, which needs to be explored.
